# Supplementary material for: The interactome of intact mitochondria by cross-linking mass spectrometry provides evidence for coexisting respiratory supercomplexes
Source: Mol Cell Proteomics. 2017 Dec 8;17(2):216–32. doi: 10.1074/mcp.RA117.000470 (PMC5795388; doi:10.1074/mcp.RA117.000470)
Supplement: Supplemental Data [file supp_RA117.000470_133922_0_supp_23978_fzffwf.zip › spectra_annotation/mito_DR_spectra_annotation/482-2-3-1-7-1.pdf]

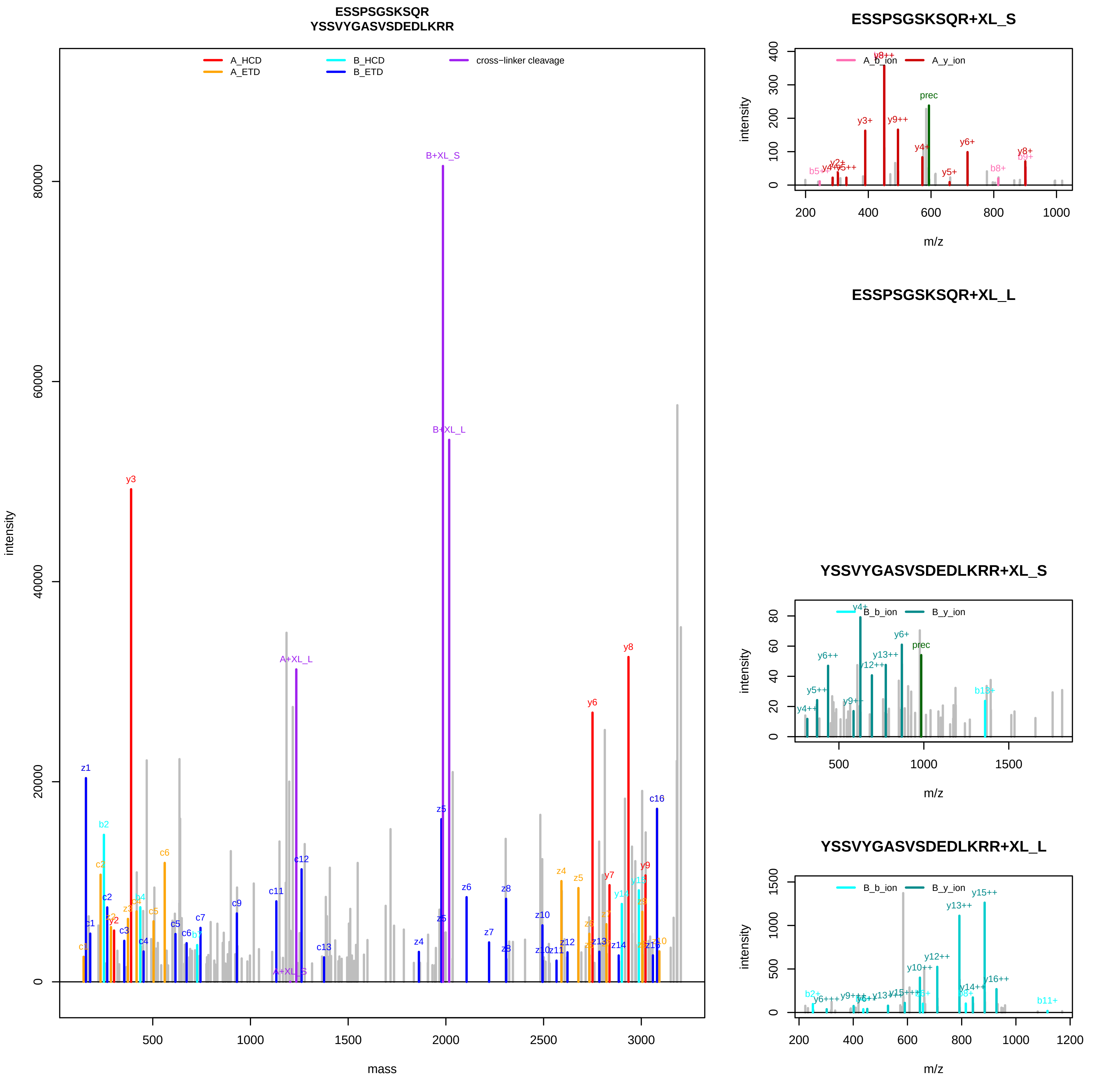
ESSPSGSKSQR
YSSVYGASVSDEDLKRR
A_HCD
A_ETD
B_HCD
B_ETD
cross−linker cleavage
intensity
mass
0
20000
40000
60000
80000
500
1000
1500
2000
2500
3000
B+XL_S
B+XL_L
A+XL_L
A+XL_S
y3
y6
y8
y7
y9
c16
c12
c11
c9
c7
z1
b2
c6
z6
z7
z8
z10
z12
z4
z5
y14
ESSPSGSKSQR+XL_S
A_b_ion
A_y_ion
prec
y8++
y3+
y9++
y4+
y6+
y5+
b8+
y8+
b9+
b5++
m/z
200
400
600
800
1000
0
100
200
300
400
ESSPSGSKSQR+XL_L
YSSVYGASVSDEDLKRR+XL_S
B_b_ion
B_y_ion
y4+
y6+
y6++
y13++
y12++
y5++
y9++
y4++
b11+
1500
20
40
60
80
YSSVYGASVSDEDLKRR+XL_L
y15++
y13++
y12++
y10++
y16++
y14++
b11+
b2+
1200
500
1000
